# Basic understanding of posterior probability

**DOI:** 10.3389/fpsyg.2015.00680

**Published:** 2015-05-22

**Authors:** Vittorio Girotto, Stefania Pighin

**Affiliations:** Center for Experimental Research on Management and Economics, Department of Culture Project, University IUAV of VeniceVenice, Italy

**Keywords:** posterior probability, updating, number of chances, natural frequencies, intuitive judgments

Consider the following task

[Task A]

A prenatal test determines whether an unborn child has a chromosomal anomaly. A priori, namely, before undergoing the test, a pregnant woman has a 4% chance of having a child with the anomaly. If a woman has a child with the anomaly, there is a 75% chance that she has a positive test result. If she does not have a child with the anomaly, there is still a 12.5% chance that she has a positive test result. Emma, a pregnant woman, undergoes a prenatal test. The result is positive. What is the probability that she has a child with the anomaly?

To answer correctly, one has to integrate the prior probability that a woman has a child with the anomaly (i.e., the prevalence rate: 4%) with information about the test's statistical properties. On the basis of this information and the evidence that Emma tested positive, one can produce a correct posterior evaluation by computing the ratio:

Probability (Anomaly|Positive Test Result) = Probability (“Positive Test Result and Anomaly”)/Probability (“Positive Test Result”).

To obtain the numerator, one has to combine the prevalence rate and the test's sensitivity rate (i.e., 4% × 75% = 3%). To obtain the denominator, one has to combine the complement of the prevalence rate and the false positive rate (i.e., 96% × 12.5% = 12%), and then add it to the initially obtained value (i.e., 3% + 12% = 15%). Very few respondents, including health-care professionals, produce the correct probability ratio (i.e., 3%/15% = 20%). Failures to solve tasks of this sort lead to pessimistic conclusions about naive probabilistic reasoning (e.g., Casscells et al., 1978). Subsequent studies, however, licensed more optimistic conclusions, showing that some versions of these tasks led to better performances. About half of the respondents succeed when reasoning with natural frequencies (e.g., “Three out of the 4 women who had a child with the anomaly had a positive test result”) or numbers of chances (e.g., “In 3 out of the 4 chances of having a child with the anomaly the test result is positive”; see, respectively, Hoffrage and Gigerenzer, 1998; Girotto and Gonzalez, 2001). On the basis of these results, the current, common account is that posterior probability reasoning improves in versions that allow respondents to both rely on an appropriate representation of subsets of countable elements (e.g., observations, tokens), and to easily associate posterior evidence with one of these subsets (Barbey and Sloman, 2007).

A generally unnoticed aspect of the results mentioned above is that they concern educated respondents, like undergraduates and physicians, and that only about half of these respondents benefit from the simplified versions of the tasks. Even more unnoticed is the fact that respondents sampled from the general public do not benefit at all from these versions. Indeed, in samples of pregnant women, many of whom had a high school level of education or less, almost all respondents failed to compute the correct probability ratio, even if they had to reason about natural frequencies (Bramwell et al., 2006) or numbers of cases (Pighin et al., 2015). In other words, they failed tasks that, in principle, should have activated the appropriate set representation. Their failure is striking because, unlike the participants of previous studies who had to reason about hypothetical scenarios, these women reasoned about realistic prenatal test results, and were personally interested in understanding them correctly.

In sum, contrary to the common account, naive respondents do not perform well on tasks devised to improve their understanding of posterior probability. These tasks mimic everyday problems, like calculating the post-test probability of diseases. However, they are unlikely to be the best tools to investigate whether naive respondents possess a basic intuition of posterior probability, and whether they are able to update their evaluations in the light of new evidence (Girotto and Gonzalez, 2007). Indeed, these tasks do not require respondents to revise any initial judgment (Girotto and Gonzalez, 2008; Mandel, 2014). Rather, they simply ask for only one judgment on the basis of various pieces of evidence (e.g., the prevalence rate, the result of the test and its statistical properties). Moreover, these verbal tasks convey numerical information by means of symbols and require an explicit numerical evaluation. Therefore, they can be employed only with literate respondents who have acquired a numerical symbolic system. Producing an explicit numerical estimation in numbers or words, however, is not the only way in which individuals may assess chance. Consider the following task:

[Task B]

Respondents are presented with a box containing five red chips (four round and one square) and three green chips (all square). The experimenter says, “I will take one chip out of the box without looking inside. Do you think that I will get a red or a green chip?”

Unlike Task A, and other verbal tasks used in adult Western literature, Task B does not convey probabilities by means of numerical symbols, and does not require respondents to produce an explicit numerical evaluation. Rather, it presents a set of tokens, and asks for a qualitative judgment or choice between two outcomes that may occur by taking one token out of the set at random (i.e., drawing a red vs. a green chip). To produce a suitable answer, respondents can reason extensionally, by considering and comparing the ways in which the outcomes may occur (Johnson-Laird et al., 1999). Accordingly, respondents will predict the occurrence of the outcome that may be produced in more ways (i.e., drawing a red chip). Numerate respondents could make a precise enumeration of the chances favoring each outcome (e.g., “There are 5 chances of drawing a red chip vs. 3 chances of drawing a green chip”). On this basis, they could even produce an explicit and correct absolute evaluation (e.g., “There are 5 chances out of 8 of drawing a red chip”). Of course, non-numerate respondents could not do so. However, the ability to make approximate comparisons of quantities emerges before (e.g., Barth et al., 2005) and without schooling (e.g., Pica et al., 2004). Therefore, even individuals who lack any formal numerical knowledge should produce suitable predictions in simple tasks like Task B. Indeed, both Western 5-year-olds (e.g., Davies, 1965; Girotto and Gonzalez, 2008) and preliterate Mayan adults (Fontanari et al., 2014) answer “red,” that is, they choose the more likely outcome, and they do so even when they have to consider large sets of tokens. In sum, non-numerate individuals are able to compare the chances of two competing outcomes, without being able to express them numerically, and without necessarily making an explicit and precise counting of the number of chances favoring each of them.

Notably, these individuals also revise their evaluations on the basis of a new piece of evidence:

[Task B']

Upon the completion of Task B, the experimenter say, “I have taken one chip out of the box. I have it in my hand and I feel that it is square. Do you think that I got a red or a green chip?

To choose the more likely outcome (“green”), respondents should focus on the subset of possibilities compatible with the evidence (the four squares). Five-year-olds do so, updating their initial judgments and choices suitably (Girotto and Gonzalez, 2008/Studies 1 and 2). They succeed even in tasks that imply more complex combinations of prior and posterior information (Bonawitz et al., 2013), or reasoning about a single, non-repeatable event produced by an intentional agent (Girotto and Gonzalez, 2008/Study 3). Fontanari et al. (2014) have extended these results by presenting preliterate Mayan adults with the same sort of tasks. Despite their lack of any sort of formal education, these respondents performed like Western controls, revising their initial choices in the light of new evidence. Finally, measures of looking times suggest that even preverbal infants form rational expectations about uncertain events by integrating different sources of information in a coherent way (Teglas et al., 2011). Together, these findings corroborate the view that, along with the application of non-extensional heuristics (Tversky and Kahneman, 1974), naive reasoning about probabilities often relies on extensional procedures: respondents infer the probability of an event from the various ways in which it could occur (Johnson-Laird et al., 1999).

Two notes are in order about the tasks that have documented the existence of an early understanding of prior and posterior probability (e.g., Task B and B'). First, these tasks are not natural frequency tasks. Indeed, they do not convey natural frequency information and do not ask for a frequency prediction. The following one is an example of a proper natural frequency task:

[Task C]

The experimenter says, “This box contains some chips. You do not know their colors. You observe me drawing a chip at random from the box, and replacing it in the box 8 times. My sample shows 5 red and 3 green chips. I'll draw a chip at random 8 more times. Do you think that the new sample will show more red or more green chips?”

Task C is apparently similar to Task B. In both cases, one can answer by considering sets of countable elements (i.e., prior possibilities and actual frequencies, respectively), and by making a similar comparison (i.e., 5 red chips vs. 3 green chips, and 5 draws of a red chip vs. 3 draws of a green chip, respectively). The two answers, however, cannot be assimilated. In Task B, one reasons about a set of prior possibilities before making any actual experience. In Task C, one reasons about a set of observations gathered through a “natural sampling,” which is “the process of encountering instances in a population sequentially. The outcome of natural sampling is natural frequencies” (Gigerenzer and Hoffrage, 1999, p. 425).

Second, tasks that do not ask for an explicit numerical evaluation, including those that imply reasoning about few possibilities, do not guarantee correct performance neither in children nor in adults (Nickerson, 1996; Johnson-Laird et al., 1999). Consider, for example, Task B. Young children succeed in it, basing their answer on prior possibilities (e.g., “You will get a red chip because there are more red than green chips”). However, if one transforms Task B into a frequency-like task, they fail. In other words, if one makes a series of random draws from the same box, and asks young children to make a prediction for each of them, they tend to use erroneous strategies like “Predict the color that was not predicted in the previous trial” (Brainerd, 1981; Teglas et al., 2007/Studies 3 and 4). It should be noted that even literate adults make erroneous predictions in situations in which they have to extract frequencies from actual observations rather than to process numerical symbols. For example, they fail versions of Task A in which they are presented with a series of medical records, each representing a patient, his/her health condition and the presence/absence of a given symptom (e.g., Gluck and Bower, 1988). Along with the finding that young children can reason correctly about events before experiencing their actual frequency, the finding that literate adults err in experience-based reasoning tasks is difficult to explain following the hypothesis that the human mind is “developmentally and evolutionary prepared to handle natural frequencies” (Gigerenzer and Hoffrage, 1999, p. 430).

In conclusion, even literate adults have difficulties in producing correct posterior evaluations. They appear to be unable to combine prior information and new evidence in a normative way in tasks whose solution depends on the combination of numerical values, including tasks that have been devised to improve posterior probability reasoning. However, recent studies have shown that even young children and preliterate adults can succeed in tasks whose solution depend on a simple comparison of possibilities. In sum, naive individuals possess correct intuitions of prior and posterior probabilities, and such intuitions emerge early in the course of development and regardless of culture and education.

## Conflict of interest statement

The authors declare that the research was conducted in the absence of any commercial or financial relationships that could be construed as a potential conflict of interest.
